# Commentary on “Clinicopathological features of programmed cell death-ligand 1 expression in patients with oral squamous cell carcinoma”

**DOI:** 10.1515/med-2022-0431

**Published:** 2022-02-01

**Authors:** Juan Francisco Peña-Cardelles, José Ernesto Moro-Rodríguez, José Luís Cebrián-Carretero, José Juan Pozo-Kreilinger

**Affiliations:** Oral surgery and Implantology Department, Universidad Rey Juan Carlos, Faculty of Health Sciences, University Clinic, Av. de Atenas, S/N, 28922 Alcorcón, Madrid, Spain; Oral and Maxillofacial Surgery Division and Prosthodontics Division, School of Dental Medicine, University of Connecticut, Connecticut, United States; Universidad Rey Juan Carlos, Madrid, Spain; Oral and Maxillofacial Surgery Department, Hospital Universitario La Paz, Madrid, Spain; Department of Pathology, Universidad Autónoma de Madrid, Madrid, Spain; Department of Pathology, Hospital Universitario La Paz, Madrid, Spain

The clinical and pathological value of programmed cell death-ligand 1 (PD-L1) expression in the oral squamous cell carcinoma (OSCC) has originated the research of different studies that try to determine the real relationship between this disease and this immune checkpoint since the latest clinical trials have related possible improvements in the blockade of this immune checkpoint through immunotherapy.

Is for that reason that new systematic reviews have tried to explain this relationship. Cui and Su (2020) in the review and meta-analysis “Clinicopathological features of programmed cell death-ligand 1 expression in patients with oral squamous cell carcinoma” aimed to clarify the role of PD-L1 in OSCC [1]. For that, they included a large sample of 1,947 participants from a total of 15 studies.

The authors found very interesting results concerning PD-L1 and its correlation with sex, stages, and human papillomavirus (HPV) infection status, although they have described that there is a controversy between the included studies. However, the results concluded in this study may be confusing due to the large sample size of the studies included in this systematic review and meta-analysis.

The authors have used as inclusion criteria only OSCC patients confirmed by histology. However, from the total of 15 studies included, 6 studies (De Meulenaere et al., 2017; Kim et al., 2016; Sato et al., 2019, Upko et al., 2013, Hong et al, 2016; Hong et al., 2019) [2,3,4,5,6,7,8] were research carried out exclusively on patients with oropharyngeal squamous cell carcinoma (OPSCC) and another study (Ock et al., 2016) [9] included both oropharyngeal and non-oropharyngeal carcinoma. Therefore, approximately 915 patients of the total 1,947 are OPSCC patients and not OSCC.

Nowadays, it is established that OPSCC is different in comparison with OSCC, since the diagnostic, the clinical behaviour, the treatment, and the prognostic are very different, a fact that has already been described in the TNM classification [10]. Besides, this bias has already been found in other systematic reviews in this field [11].

On the other hand, the authors have found a strong relationship between the HPV prevalence and OSCC, obtaining important conclusions concerning the HPV status and the PD-L1 in OSCC. Nevertheless, almost the total sample where the HPV was studied was compounded by OPSCC studies, since other studies have demonstrated the highest prevalence in OPSCC [12], while in the OSCC it is almost not prevalent [13], which is not consistent with the data and outcomes obtained with respect to the HPV status in the Cu and Su meta-analysis.

Due to the above-mentioned, we consider that it must have a caution interpretation to OSCC concerning the outcomes obtained by Cu and Su since almost half of the sample are OPSCC participants.
